# Understanding the Adoption and Diffusion of a Telemonitoring Solution in Gestational Diabetes Mellitus: Qualitative Study

**DOI:** 10.2196/13661

**Published:** 2019-11-28

**Authors:** Carine Khalil

**Affiliations:** 1 Paris Descartes University Paris France

**Keywords:** gestational diabetes, telemonitoring, diffusion of innovation theory, qualitative research

## Abstract

**Background:**

Women with gestational diabetes mellitus (GDM) require regular follow-ups and overall management to normalize maternal blood glucose and improve pregnancy outcomes. With the advancements made in the digital field, telemedicine is gaining popularity over traditional health care approaches in different medical fields. As for GDM, telemonitoring solutions seem to improve women’s quality of life and enhance self-management.

**Objective:**

The aim of this study is to understand, from patients’ and health care professionals’ (HCPs) perspectives, what drives the adoption and diffusion of a telemonitoring solution (myDiabby) in a context where telemonitoring activities are still not compensated like traditional follow-ups.

**Methods:**

The study was conducted in 12 diabetes services in France using myDiabby for monitoring and managing patients with GDM. A qualitative research approach was adopted for collecting and analyzing data. A total of 20 semistructured interviews were conducted with HCPs working in different health structures in France, and 15 semistructured interviews were conducted with patients who had been using myDiabby. Data were analyzed using a thematic analysis approach.

**Results:**

Different determinants need to be taken into consideration when adopting an innovative health technology. By drawing on the diffusion of innovation theory, a set of factors associated with the technology (the relative advantages, compatibility, ease of use, testability, and observability of the telemedicine platform) has been identified as affecting the adoption and diffusion of telemonitoring solutions in French diabetes services. In addition, data analysis shows a set of environmental factors (the demographic situation of HCPs, the health care access in rural communities, and the economic and political context in France) that also influences the spread and adoption of telemonitoring systems in French hospitals.

**Conclusions:**

Even though telemonitoring activities are still not remunerated as traditional follow-ups, many French HCPs support and encourage the adoption of telemonitoring systems in GDM. As for patients, telemonitoring systems are perceived as a useful and easy way to monitor their GDM. This study contributes to recognizing the value of telemonitoring interventions in managing GDM and considering the expansion of telemonitoring to other chronic conditions.

## Introduction

### Background

Gestational diabetes mellitus (GDM) is defined as glucose intolerance first recognized during pregnancy [1]. The increasing prevalence of obesity and the advanced maternal age seem to increase the number of women with GDM [2,3]. This prevalence varies depending on the screening criteria; GDM is estimated to occur during 6% to 15% of pregnancies [4]. According to the new criteria defined by the International Association of Diabetes and Pregnancy Study Groups, GDM is estimated to occur in 14% pregnancies in France and represents one of the most frequent pathologies during pregnancy [5].

Many perinatal and postpartum complications are associated with GDM [6,7]. Adverse outcomes include the development of type 2 diabetes and cardiovascular disease in the mothers, preterm delivery, shoulder dystocia, stillbirths, clinical neonatal hypoglycemia, hyperbilirubinemia, and cesarean deliveries [3,6,8]. The World Health Organization recommends universal screening of all women for GDM at 24 to 28 week’s gestation [9]. Women with GDM require regular follow-ups and overall management to normalize maternal blood glucose and improve pregnancy outcomes [10-13]. However, an intensive surveillance in GDM can be costly and labor intensive [3]. It represents a real burden for health professionals and patients. Owing to the increasing number of women with GDM requiring regular follow-ups, along with the shortage of health care professionals (HCPs), telemedicine interventions can reduce outpatient clinic visits and provide better overall management for patients with GDM [14,15]. With the advancements made in the digital domain, telemedicine is gaining popularity over traditional health care approaches in different medical fields. Although a growing number of research studies highlighted the value of telemonitoring for managing diabetes and addressed its feasibility and acceptability among health professionals and patients, results are still modest. Besides, few studies have used qualitative approaches to examine in-depth factors that influence the adoption and diffusion of telemonitoring solutions in French health establishments and among patients.

Therefore, this study aimed at understanding, from patients’ and HCPs’ perspective, what drives the adoption and diffusion of myDiabby (a telemonitoring platform) in French health care centers where telemonitoring of women with GDM is still not compensated.

### Telemedicine in Gestational Diabetes: Related Work

Some research studies focused on examining the feasibility and the acceptance of telemedicine systems for managing GDM [16-20]. The literature review shows a high degree of acceptance of telemedicine interventions in GDM. The use of telemedicine seems to improve patient satisfaction regarding access to care [19,21], reduce the need for outpatient clinic visits [16,19], and enhance patient-caregiver information exchanges [22]. In addition, the use of telemedicine solutions seems to increase the efficacy of health care providers [21,23,24]. It also seems to improve patients’ self-efficacy in managing their diabetes [16,25] and is cost saving [26]. Patients with GDM feel supported with telemedicine solutions and are willing to use them again [17,27].

Despite the underlined acceptance and satisfaction regarding the use of telemonitoring systems in GDM, factors that drive their adoption and diffusion in health establishments are less investigated. Previous studies mainly focused on telemedicine outcomes (feasibility, satisfaction, and clinical outcomes) without providing an in-depth analysis of how telemonitoring systems are adopted and diffused, particularly in France. In this respect, this study will draw on the diffusion of innovation theory to examine factors that drive the adoption and diffusion of myDiabby in French health care centers where telemonitoring activities are still not remunerated.

### The Diffusion of Innovation Theory

The diffusion of innovation theory offers an appropriate lens for examining factors that influence the adoption and diffusion of an innovation in specific context settings. An innovation is defined as *an idea, practice, or project that is perceived as new by an individual or other unit of adoption* [28]. Therefore, it is not necessarily invented recently. If individuals perceive it as new, then it is still an innovation for them. Rogers [28] highlighted 5 key attributes of an innovation that influence its likelihood of adoption and diffusion in a specific context. These attributes are relative advantage, perceived compatibility, complexity, trialability, and observability. Relative advantage is the extent to which an innovation is perceived better than the idea it supersedes [28]. Innovations are adopted when users perceive them as a better option than the ones they currently have or use. Perceived compatibility is the degree to which an innovation is perceived by the potential adopters to be consistent with their existing values and current needs. In other words, an innovation has a greater chance to be adopted and diffused when it is aligned with the cultural norms and adopters’ needs. Complexity is the extent to which an innovation is perceived to be difficult to understand and use [28]. This attribute is also found in the technology acceptance model under the perceived ease of use. Innovations that are difficult to use will be adopted more slowly than the ones that are perceived to be less difficult and complicated. A high degree of complexity can lead to a high degree of frustration among potential adopters. Trialability is the possibility to experiment and test an innovation before committing to it. Innovations with higher trialability are more likely to be adopted by individuals [29]. They allow the potential user to try out an innovation and return to pre-existing situation without much cost. Finally, observability is the degree to which the results of an innovation are visible to potential users. High visibility and demonstrability of the benefits of an innovation encourage more individuals to adopt it [29].

According to Rogers and Singhal [28], other determinants also influence the spread and the adoption rate of an innovation: the communication channels, the social system, and the characteristics of the adopters. The communication channels are crucial to diffuse the information about the perceived advantages of an innovation. They can create or change people’s attitudes toward an innovation. Communication channels can include any mean (newspaper, television, reports, and intrapersonal communication) through which people diffuse and obtain information about the innovation and its benefits. As for the social system, it is defined as a “set of interrelated units engaged in joint problem-solving to accomplish a common goal.” The social system affects individuals’ attitudes toward an innovation. As for the characteristics of the adopters, individuals can be categorized into 5 groups: innovators (first group to adopt an innovation), early adopters, earlier majority, later majority, and laggards (the strongest resisters to the adoption of an innovation).

Even though previous studies have used the diffusion of innovation theory as a theoretical lens to examine the adoption of health care information technologies [30,31], these studies rarely addressed the context of GDM [32], even less in France. Therefore, this study used the diffusion of innovation theory to understand what drives French hospitals to adopt and diffuse myDiabby.

## Methods

### Research Settings

myDiabby health care is a telemonitoring platform that offers a new solution for monitoring and managing GDM. It replaces paper diary records and allows patients to manually enter their blood sugar level in the system or have their data transferred directly from the glucometer to the app (via Bluetooth). myDiabby includes a color coding (green, orange, and red) that helps patients understand their blood glucose concentration. Patients can also enter their dietary records and privately share their concern(s) and question(s) with their health care team. As for HCPs, myDiabby allows them to telemonitor their patients’ data (blood glucose level) via a customized alert system, adjust or prescribe insulin doses, and privately chat with their patients. myDiabby is implemented in 230 French health centers. A total of 360 new patients are telemonitored via myDiabby every month.

In general, patients diagnosed with GDM are invited to a therapeutic education session in the health center of their region. During this session, the health care team (the diabetes specialist, the midwife, and the nurses) meets with the patients, provides them with explanations regarding their pathology, and answers their questions and concerns. The therapeutic education session is also the occasion to raise patients’ awareness regarding the need for self-monitoring and managing their diabetes. During this session, the health care team provides their patients with a glucometer, introduces them to the myDiabby health care platform, and gives them secured personal access to the telemonitoring platform. Patients are supposed to test and enter their blood sugar level 6 times per day (before and after breakfast, before and after lunch, and before and after dinner). However, those with stable blood glucose levels can decrease their test and data entry to 3 times per day. Although some patients prefer entering their data manually to be more aware of their results, others do it manually, especially when their Bluetooth does not work properly.

Nevertheless, the use of myDiabby has not eliminated phone interactions. Health care team members still contact their patients (via phone) to follow-up with those who have not entered their data for 2 days in a row or to discuss changes in their treatment decision.

### Data Collection

The qualitative research was conducted in 12 diabetes services in France using myDiabby platform for monitoring and managing patients with GDM. Among the 12 diabetes services, 11 are attached to public health centers and 1 is a private diabetes clinic. Each diabetes service has its own organization, coordination procedures, patient education process, and telemonitoring protocol. HCPs do not follow any standardized protocol for viewing patients’ data in the system and responding to them via the app. They have developed their own “practice” for telemonitoring patients and managing their GDM.

Data collection took place from January 2018 to May 2018. A set of semistructured interviews was performed with patients and health professionals working in 12 different health care centers in France.

On the one side, interviews were scheduled with HCPs to understand in depth their perspectives regarding the adoption and diffusion of myDiabby. The sample includes HCPs working in French diabetes services (public or private) and having experience in both traditional follow-ups and telemonitoring. Therefore, 32 HCPs from 18 different diabetes services that use myDiabby and other telemonitoring platforms were contacted. Personalized emails were sent to them, explaining the purpose of the study and inviting them to participate in the study. A total of 20 HCPs (8 diabetes specialists, 8 educational nurses, 2 dietitians, 1 gynecologist, and 1 midwife) from the 12 diabetes services showed interest in participating in the study and agreed on being contacted. A total of 5 HCPs did not respond to the emails, and 7 highlighted their lack of availability. In all, 20 participants gave their oral consent to be interviewed. Interviews lasted between 35 and 45 min. A saturation in the gathered data was reached after 20 interviews.

On the other side, interviews were also scheduled with a convenience sample of patients who have previously had or currently have GDM. The sample included women who have been diagnosed with GDM and have used a glucometer and myDiabby during at least 1 pregnancy. The decision of including pregnant women with active GDM and women who already delivered helped increase the sample and gain more insights into their perceptions of the telemonitoring platform. Women who were diagnosed with diabetes before their pregnancy were excluded from the study.

Women with GDM were identified from the list of patients of HCPs who had already been interviewed. Given the nature of this study and the no risks associated, this research did not require an Institutional Review Board approval (according to the French law “JARDE”). However, the purpose of the research was explained to patients before getting their approval and oral consent to participate in the study. HCPs elucidated the reasons why these patients were asked to be part of the study, the possible discomforts they may feel during the interview, and their possibility to pass on answering any question or to even quit the conversation. They also made it perfectly clear that they were not part of the research team and that they had no interest in it. This way, patients did not feel pressured to participate. In total, 15 patients agreed on participating in the study and gave their verbal consent. Interviews lasted an average of 30 min and were recorded with the consent of the participants.

### Data Analysis

Before starting the analysis, transcripts were translated to English by a third party. For analyzing the collected data, we adopted an interpretive approach. We began with multiple readings of the transcribed interviews to understand the context and projects in which the respondents were involved. Although we adopted an open-coding approach to identify key categories in each transcribed word, sentence, or paragraph, Rogers’ theoretical lens guided us in analyzing the data. Early descriptive codes were identified with little or no inference beyond the piece of data. Therefore, data were summarized and segmented. A set of deductive themes, such as “relative advantage of myDiabby,” “compatibility of myDiabby,” and “ease of use of myDiabby,” has been identified and justified verbatim. Besides, more advanced codes or pattern codes emerged inductively, such as “the impact of the demographic context of French health care professionals” and “the context of pregnancy.” Themes were defined and justified verbatim [33,34]. The coding process was done by a coder who has expertise in qualitative research. However, the same data have been analyzed twice in an interval of 7 months. This helped compare the results and evaluate their consistency over time.

## Results

### Relative Advantage of myDiabby

#### From Health Care Professionals’ Perspective

According to the interviewees, the use of myDiabby for monitoring GDM offers a set of significant advantages compared with traditional follow-ups.

The use of myDiabby enabled regular follow-ups and improved patient care by being more reactive:

Today they [patients] have closer follow-ups.

Diabetes specialist_4

We used to have random follow-ups whereas now we are way more reactive. We put them under treatment sooner.

Diabetes specialist_1

Regular follow-ups seemed beneficial for controlling women’s glycemic level and weight:

Frankly, we have a better follow-up now. Plus, with the hypoglycemic and hyperglycemic alerts, we can directly identify patients that need to be taken in charge immediately.

Nurse_5

Women has less tendency to gain weight as the follow-up is more frequent.

Diabetes specialist_1

Women have also lost weight due to regular follow-ups.

Dietician_2

In addition, the use of myDiabby empowered women to self-manage their GDM:

myDiabby helped patients self-manage their health.

Nurse_2

Therefore, patients spent less time commuting and waiting in doctors’ offices to show their glucose level:

Patients commute less. And, we barely see those who have stable blood glucose levels.

Gynecologist_1

Patients spend less hours waiting in our office

Nurse_2

In addition, telemonitoring brought HCPs closer to patients. According to the former, myDiabby helped them interact more often with their patients:

We were really surprised with how close we felt with our patients. We got to know them better.

Nurse_1

The communication is better now. It’s more spontaneous. We have a more trustworthy relationship.

Nurse_6

Even though our participants all agreed on the relative advantages of telemonitoring, a few underlined the need of sustaining human contact with their patients, beyond their virtual desktops:

It is important to keep on seeing our patients or having them on the phone at least.

Nurse_2

myDiabby doesn’t totally replace human contact.

Diabetes specialist_6

#### From Patients’ Perspective

The use of myDiabby was perceived by patients as more reassuring and more useful than traditional follow-ups:

I knew there was always someone controlling my results, and ready to address my questions.

Patient_3

I felt safe when using myDiabby.

Patient_5

Health professionals can get my blood results in-real time, which is very useful.

Patient_2

I can directly get in contact with them and have quick answers.

Patient_6

According to the participants, myDiabby decreased the stress and anxiety related to manage their diabetes:

I knew that health providers would contact me in case my glycemic values were high.

Patient_2

It simplified my daily life and decreased my anxiety. All I had to worry about was my blood glucose test

Patient_7

It also empowered them as they felt more autonomous to self-manage their health, and it made their life easier:

Technologies help us being more autonomous. We feel more responsible.

Patient_1

I really appreciate being self-sufficient.

Patient_2

We feel more free and autonomous.

Patient_9

I don’t see myself going to the hospital every other week to control my glycemic calendar.

Patient_2

It is a real blessing to exchange virtually with health providers. I don’t have to commute.

Patient_4

That said, a few patients evoked the need of seeing their health care team when they are under insulinotherapy:

Insulinotherapy necessitates physical contact. I need to see my doctor, ask questions...I feel less reassured if I do it through distance.

Patient_1

I need to see my doctor when an insulinotherapy is required.

Patient_7

### Compatibility

#### From Health Care Professionals’ Perspective

Data analysis showed that myDiabby was consistent with HCPs’ vision and practices:

For me it doesn’t make any sense to ask these women to commute so I can check their blood glucose.

Diabetes specialist_2

Technologies are the future. We are going to use more applications and technologies very soon.

Diabetes specialist_4

We use technologies in everything now...everyone is connected in some way.

Nurse_2

#### From Patients’ Perspective

According to the patients we interviewed, technologies are becoming part of everyone’s life. Regardless of their sociodemographic background, age, and social status, almost everyone has access to the internet, downloads, and uses apps on mobile phones and tablets. Therefore, myDiabby is aligned with people’s lifestyles:

Nowadays, everyone has an iPhone and access to the Internet.

Patient_4

Therefore, the use of myDiabby or any other telemonitoring system is consistent with their way of living.

### Complexity Associated With the Use of myDiabby

#### From Health Care Professionals’ Perspective

Complexity is the extent to which an innovation is perceived to be difficult to understand and use. It can be a barrier to the adoption of a technology and its diffusion. In this study, HCPs highlighted the ease of use of myDiabby. The intuitive aspect of this telemedicine platform encouraged them to progressively adopt it and integrate it in their practices:

myDiabby is fabulous, very intuitive. I tried two different platforms that weren’t user-friendly at all. This one is very simple.

Diabetes specialist_1

The interface of myDiabby is very intuitive.

Diabetes specialist_3

It is user-friendly, easy to appropriate.

Diabetes specialist_2

#### From Patients’ Perspective

As for the patients we interviewed, they had the same opinion regarding the ease of use of myDiabby:

myDiabby is very easy.

Patient_2

Honestly I didn’t have any trouble when I started using it, it is very simple.

Patient_4

It is really easy, I can manually enter my data, modify them.

Patient_14

The perceived ease of use of myDiabby seemed to affect patients’ attitude toward telemonitoring. They seemed more inclined to use telemonitoring systems during their pregnancy.

### Trialability of myDiabby

#### From Health Care Professionals’ Perspective

Data analysis showed that the possibility to test myDiabby before committing to it had a positive impact on HCPs’ perceptions toward its adoption:

At first, our nurses were slightly reserved regarding the use of myDiabby, but once they tried it, they were very happy.

Diabetes specialist_3

When we first introduced the platform to the patients, some of them [patients] remained reserved regarding its use. But once they started using it, they were satisfied and happy.

Nurse_4

In the beginning we tried it on a few patients, and after three weeks it was adopted and used by almost everyone.

Nurse_6

In addition, HCPs were more likely to adopt the platform as they were able to customize it according to their needs:

We are able to customize it. It’s a real pleasure.

Diabetes specialist_1

The development team is very responsive and reactive. myDiabby evolves according to our requirements.

Gynecologist_1

#### From Patients’ Perspective

The patients we interviewed were given the chance to choose between myDiabby and traditional follow-ups. After attending the educational workshop and getting secure access to myDiabby, patients were able to try myDiabby and decide whether to continue using it. Their positive experience with it seems to encourage its adoption and use:

I remember the first times I had to enter my data after each glucose test... I thought it was going to be complicated and that I should go back to the paper diary records. but rapidly I got used to it... if I’m ever pregnant again and have diabetes, I would ask to be telemonitored.

Patient _5

### Observability

#### From Health Care Professionals’ Perspective

The observed benefits have encouraged other health specialists to adopt myDiabby:

Midwives are now interested in using myDiabby. It is starting to expand progressively within our structure.

Diabetes specialist_3

Our gynaecologists are now using myDiabby. They leave us messages on patients’ weight or any related health problems.

Diabetes specialist_1

In addition, the participants emphasized the impact of the observed benefits on patients’ attitudes regarding the adoption of myDiabby:

Our patients are willing to use it again.

Nurse_2

Some of them [patients] would never go back to traditional follow-ups.

Nurse_6

During their second pregnancy our patients use the application without even questioning it.

Nurse_7

#### From Patients’ Perspective

Patients expressed their tendency to share their positive feedback with their surroundings:

I was talking to my sister about how simple it was to be telemonitored.

Patient_15

I would encourage my friend to use it if she had to...

Patient_12

Beyond these factors that are directly associated with the telemonitoring technology, environmental factors seem to play an important role in encouraging telemonitoring over traditional methods: the demographic context of French health professionals, the geographic context, the pregnancy context, and the economic and political context.

### The Demographic Context of Health Professionals

In France, the number of HCPs working in diabetes care is disproportional to the number of patients with GDM. Therefore, the implementation of innovative ideas for managing patients with GDM is encouraged. According to our participants, telemonitoring helped them address this issue:

We have a mismatch between the number of patients with gestational diabetes and the number of health care professionals. We had 580 patients with gestational diabetes in 2017.

Diabetes specialist_2

We had to find another way to take care of our patients, otherwise, we wouldn’t be able to meet patients’ needs.

Diabetes specialist_8

### The Geographic Context and Health Care Access

Telemedicine seemed useful for patients living in rural areas. As mentioned previously, many patients have long hours of commuting to visit their HCPs and show their blood glucose values. Therefore, commuting is “exhausting,” especially for pregnant women. For this reason, women tend to prefer virtual follow-ups over traditional ones:

Some of our patients drive 100 kilometers in order to come to the hospital.

Diabetes specialist_3

### The Context of Pregnancy

Pregnancy can be critical for patients with GDM. In this respect, telemonitoring is helpful, as it enables regular follow-ups and encourages women to self-monitor their diabetes during their pregnancy (which is usually limited to a few months). Besides, women feel more responsible in the context of pregnancy, as their baby is also impacted by their medical condition:

Pregnancy is the ideal context; women have three months to self-manage their diabetes. Hence, they will invest themselves and do whatever it takes to protect their baby.

Diabetes specialist_8

### The Economic and Political Context

Despite the positive feedback regarding myDiabby, the participants (mostly HCPs) reported a few barriers that constrain the diffusion of telemonitoring solutions in French health centers. As telemonitoring of GDM is still not recognized as a medical “practice” in France, HCPs are unable to dedicate specific hours for telemonitoring activities. They do it for free, in addition to their work:

We do it during our working time. I do it sometimes at my house at 10pm.

Diabetes specialist_4

It is not recognized as a medical act and it is not included in our job description.

Nurse_4

Given this situation, many health professionals do not use it despite the perceived advantages:

It decreases the number of medical acts that can be billed.

Diabetes specialist_5

Liberal doctors prefer earning money which is understandable.

Midwife_1

The actual care policy emphasizes money over quality of care.

Nurse_2

However, even though telemonitoring is still not valued and recognized as a medical “practice,” many HCPs still encourage its adoption. Some of them have even suggested the expansion of myDiabby to diabetes 1 and 2:

It can be beneficial in diabetes 1 and 2 as it happens that we don’t see some patients for 5 years.

Diabetes specialist_7

It is probably interesting to use mydiabby during a specific period—after a surgery for instance—and on specific profiles.

Gynecologist_1

## Discussion

### Principal Findings

The findings underline patients’ and HCPs’ preferences for virtual follow-ups over traditional ones. The relative advantages, perceived ease of use, observed benefits, trialability, and compatibility of myDiabby with participants’ vision and needs have encouraged the use of this telemonitoring system for managing GDM:

If we decide to take the platform from them [patients], believe me, they [patients] will be very unhappy.

Diabetes specialist_3

It is becoming rare to have patients who prefer physical consultations only.

Diabetes specialist_6

That said, the characteristics of the adopters (innovators or early adopters) have also affected the adoption level of myDiabby. However, these participants have also tried other telemonitoring systems without diffusing them within their services. This could lead us to assume that the characteristics of the innovation itself have played a major role in encouraging its adoption.

Beyond the characteristics of myDiabby, data analysis shows that other factors have also influenced the spread and adoption of telemonitoring activities in French diabetes services. The context of having diabetes during pregnancy, the demographic situation of HCPs, the health care access in rural communities, and the economic and political context in France seem to encourage telemonitoring activities in French hospitals. Findings show that telemonitoring solutions are becoming inevitable in an environment where the number of patients with GDM is getting higher every year. Even though telemonitoring of GDM in France is still not recognized as a medical practice, HCPs seem to support it and encourage it.

### Conclusions

The diffusion of telemonitoring solutions in French health centers is still under investigation. Few papers have examined what drives French HCPs to adopt telemonitoring systems in a context where telemonitoring activities are not compensated.

This research paper highlights different determinants that should be taken into consideration when adopting a digital health solution. By drawing on the diffusion of innovation theory, the study emphasizes the innovation attributes that have encouraged the adoption and diffusion of myDiabby. However, beyond these attributes, addressed in previous papers, the research also shows the role of environmental factors in encouraging the adoption and diffusion of telemonitoring solutions. Therefore, this study offers a complementary theoretical perspective toward the adoption and diffusion of telemonitoring solutions in the context of GDM. Nevertheless, this research presents 2 major limitations. First, the number of interviews is limited. More interviews should be performed with patients and HCPs to generalize the findings. Second, participants (patients and HCPs) in this study can be classified in the “innovators” or “early adopters” category. This can constitute a potential bias in the results that are very positive. Even though the purpose of the study was to understand what motivates individuals to adopt telemonitoring systems, it would have been more interesting to include nonusers and resisters to the adoption of telemonitoring solutions.

## Abbreviations

GDM: gestational diabetes mellitus
HCP: health care professional
